# The liver fluke *Campula oblonga* from harbour porpoise *Phocoena phocoena* stranded on the Danish North Sea Coast: updated morphometric and molecular characteristics

**DOI:** 10.1007/s00436-026-08688-0

**Published:** 2026-04-30

**Authors:** Kurt Buchmann, Tim K. Jensen, Michelle L. Quaade, Per W. Kania

**Affiliations:** Department of Veterinary and Animal Sciences, Faculty of Health and Medical Sciences, University of Copenhagen, Frederiksberg C, DK-1870 Denmark

**Keywords:** Denmark, Porpoise, Parasite, Liver, Trematoda

## Abstract

One female specimen of the cetacean harbour porpoise *Phocoena Phocoena* (Linnaeus, 1758) was found dead on the North Sea coast of Denmark (Jutland) in May 2025. During necropsy severe pathological changes of the liver were observed (hepatic fibrosis, enlarged and thickened biliary duct walls) and when dissecting the biliary ducts six intact trematodes (total length 6.9–7.9 mm, width 1.9–2.0 mm) were recovered. Following conservation in formalin four parasites were haematoxylin stained, one specimen kept unstained but all five mounted for morphological analysis. The sixth specimen conserved in 70% ethanol was subjected to DNA purification and subsequent PCR and sequencing. All the parasites were identified as fully adult *Campula oblonga* (Cobbold, Trans Linnean Soc Lond 22(3):155-172, 1858). Morphometric, morphological and molecular data (NADH dehydrogenase, subunit 3 (ND3), mtDNA) are presented and indicate close relation with previous isolates from the North and Baltic Seas but a lower similarity to Pacific conspecifics.

## Introduction

Marine mammals are fully integrated elements of the marine food webs and carry a wide series of parasites transmitted by intermediate hosts in the ecosystem (Diaz-Delgado et al. 2018). This is also the case for the common cetacean *Phocoena phocoena* (Linnaeus, 1758) with the vernacular name harbour porpoise, which is commonly occurring in the Atlantic Ocean, connecting to the North and the Baltic Seas. It is well known that cestodes, trematodes and nematodes occur in various organs of this mammalian host (Rokicki et al. 1997; Gibson et al. 1998; Siebert et al. 2001; Lehnert et al. 2005; Fraija-Fernández et al. 2016; Dzido et al. 2021). Morphological descriptions have in most cases been considered sufficient for a species identification and delimitation of the isolated parasites, because the original descriptions basically were based on morphology only (Adams et al. 1998). However, molecular investigations have made valuable contributions to diagnostic precision and a few parasitological and pathological studies have mainly used molecular tools for species identification (Fernandez et al. 1998; Fraija-Fernández et al. 2016; Nakagun and Kobayashi 2020). Thus, sequencing ribosomal and mitochondrial DNA for alignments and phylogenetic analyses has facilitated further subdivisions into strains and races (Kim et al. 2025). In this study we investigate a trematode isolated from the bile ducts of *P. Phocoena*, which had been found dead on the west coast of Denmark. We consider this parasite to be *Campula oblonga* Cobbold, 1858 close to the type species (recovered in 1855 from harbour porpoise from the North Sea coast of Scotland) and originally described by Cobbold (1858). We present morphometric, morphological and molecular data supporting our species diagnosis and discuss the phylogeny of strains in the Atlantic versus the Pacific.

## Materials and methods

### Host species

A meager female specimen of harbour porpoise *P. phocoena* was found dead on May 17, 2025. Location: Corpus stranded on the beach by the Skagerrak Sea (connected to the North Sea), Tornby south of Hirtshals, Northern Jutland (coordinates Long. 9.91717681288719, Lat. 57.5590043928631). The total length of the whale was 159 cm, the body weight 53 kg, blubber layer thickness 10–15 mm. The whale was pregnant with a fetus (total length 73 cm) and with wide open birth canal. Large parts of the liver was fibrotic, with abscesses, chronic hepatitis and cholangitis characterized by ectasia, enlarged, cavernous bile ducts (up to 2 cm). Contents brownish and grainy.

## Parasites

A total of six intact trematodes were recovered from the bile ducts and transferred to a Petri dish with water. One specimen was conserved in 70% ethanol for subsequent molecular identification and five specimens were fixed in neutral formalin for subsequent morphological identification.

## Staining and slide preparation

Four of the formalin fixed trematodes were stained with haematoxylin (Mayers), the fifth was kept unstained, but all were placed on microscope slides, cleared with glycerine, and embedded in glycerine-gelatine (with a light mechanical pressure on the cover slip to flatten the specimens). Further microscopical analysis (morphometrics and morphology) was performed under the Leica Microsystems light microscopes Leica DM5000B (Germany) and Leica S9i (Switzerland) (Buchmann 2007).

**Fig. 1:**
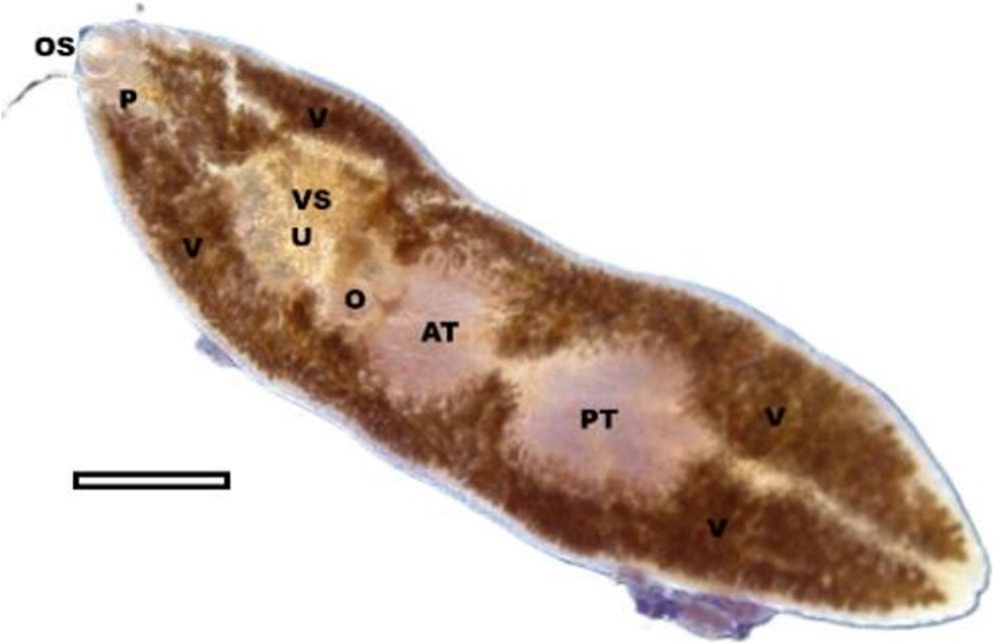
Whole mount of adult specimen of *Campula oblonga* Cobbold, 1858 isolated from *P. phocoena* in Denmark (haematoxylin stained). Trematode in toto: OS: Oral sucker, VS: Ventral sucker position (not fully visible due to egg filled uterus), P: Pharynx, U: Uterus, O: Ovary, AT: Anterior testes, PT: Posterior testes., V: Vitellaria. Scale bar: 1 mm

**Fig. 2:**
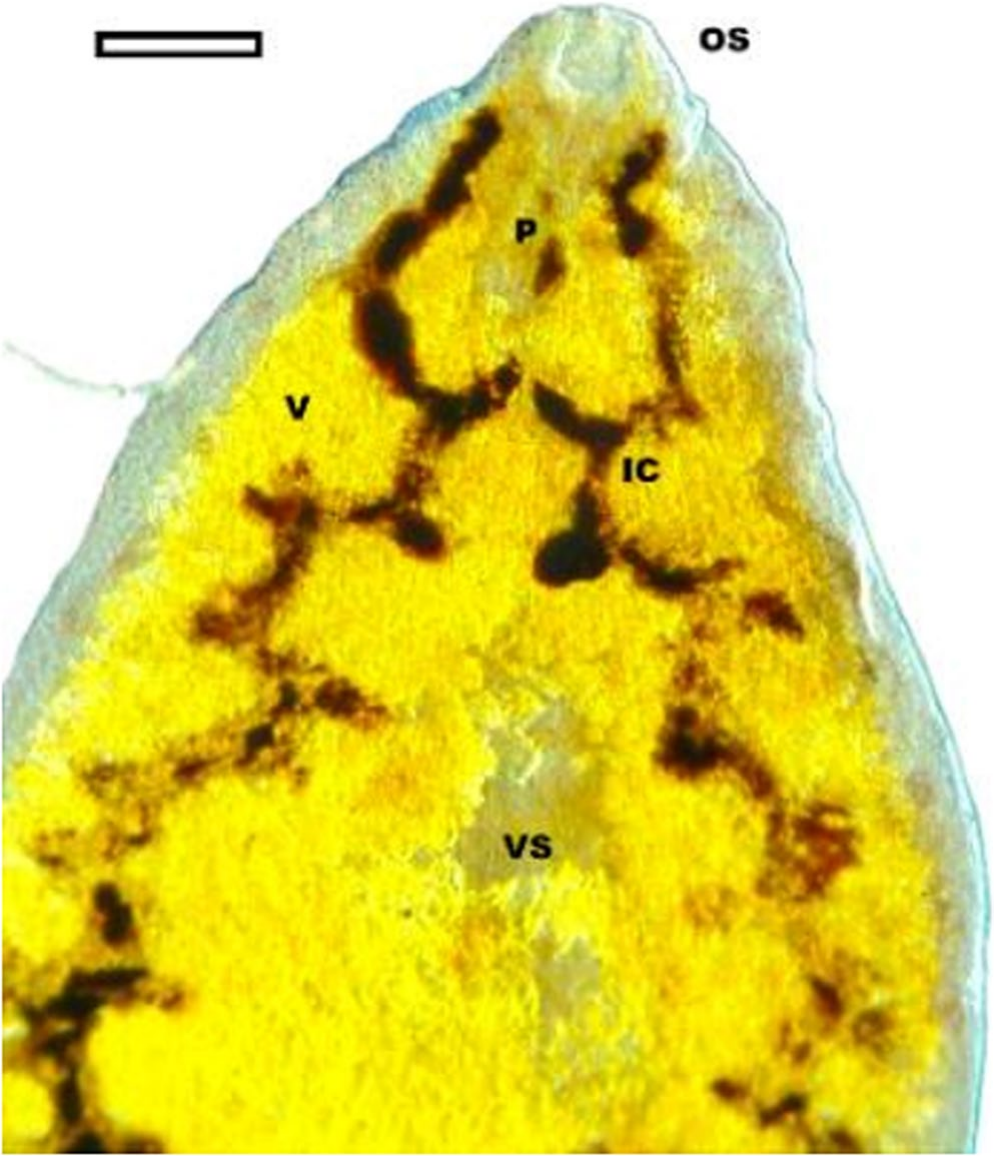
Details of anterior part of *C. oblonga*. Oral sucker and anterior branches of the intestinal caeca. OS: Oral sucker, P: Pharynx, VS: Ventral sucker position (not fully visible due to egg filled uterus), IC Intestinal caeca, V: Vitellaria. Scale bar: 350 μm

**Fig. 3:**
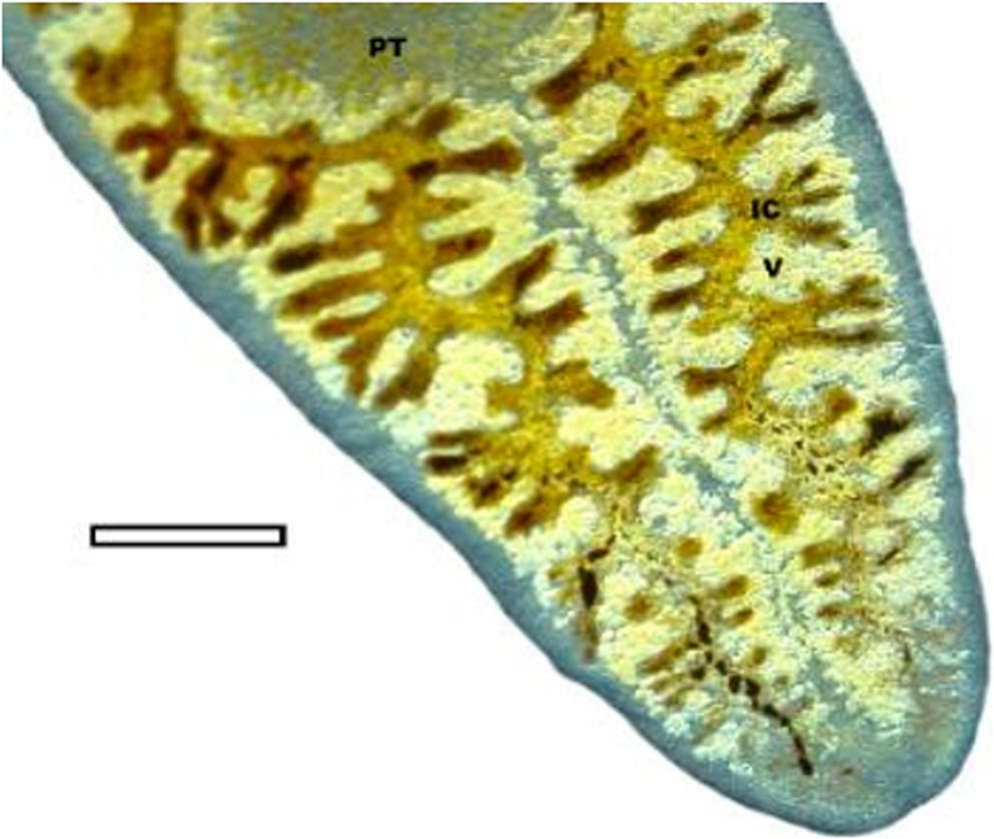
Posterior part of *C. oblonga*. Characteristic morphology of the zig-zag-shaped intestine. PT: Posterior testes, V: Vitellaria, IC: Intestinal caeca. Scale bar: 500 μm

## DNA purification

The ethanol conserved specimen was transferred to an Eppendorf tube (Axygen→, USA) and subjected to DNA-purification. Genomic DNA was purified by using the QIAamp DNA Mini Kit (cat.no. 61306, Qiagen, Denmark) according to the manufacturer’s instructions except that 50 µl instead of 200 µl elution buffer was used.

## PCR and sequencing

PCR was performed using the primers ND3F and ND3-4 (Fernández et al. 1998) amplifying the mtDNA gene encoding NADH dehydrogenase subunit 3 (ND3). This gene has proven suitable for differentiation of various genera and species within the family Brachycladiidae (Kim et al. 2025). In brief, the reactions were conducted in a BioRad T100 Thermal Cycler (cat.no. 1861096, Bio-Rad Laboratories, Denmark) in a 60 µl reaction consisting of 1 mM forward primer, 1 mM reverse primer (both from Tag Copenhagen, Denmark), 1 mM dNTP mix (dNTP Blend, cat.no. 10085714, Fisher Scientific, Denmark), 3 units DNA Polymerase (0.6 µL), 6 µL 10x Reaction buffer, 1.8 µl 50 mM MgCl2 (BIOTAQ DNA Polymerase, cat.no BIO-21060, Saveen & Werner ApS, Denmark). The PCR the conditions were pre-denaturation at 95°C for 5 min, 40 amplification cycles of denaturation at 95°C for 30 s / annealing at 54°C for 30 s / elongating at 72°C for 30 s, and post-elongation at 72°C for 7 min. PCR products were visualized using 1.5% agarose gel-electrophoresis. The forward primer was ND3 F1 (5’- GCGTTAGCAGGATCCTGTGATATAG-3’) and was the reverse primer NC2 (5’-TTAGTTTCTTTTCCTCCGCT-3’) (Fernandez et al. 1998). The product was purified by the means of the Illustra™ GFX™ PCR DNA and Gel Band Purification Kit (cat.no. 28-9034-71, VWR International A/S, Denmark) and measured in a Nanodrop 2000 spectrophotometer (Saveen & Werner ApS, Danmark).

### Sequencing and phylogenetic analysis

The products were sequenced at Macrogen Europe, Netherland, and analyzed by the software CLC- Main Workbench v20.0.4 (QIAGEN, Denmark). Maximum Likelihood Phylogeny was done using the software CLC. Alignments were constructed in Clustall W, model testing suggested that HKJY + G + T was the best model, and a phylogram was constructed by the construction method UPGMA (10 bootstraps).

## Results

### Morphology and morphometrics

**Fig. 4:**
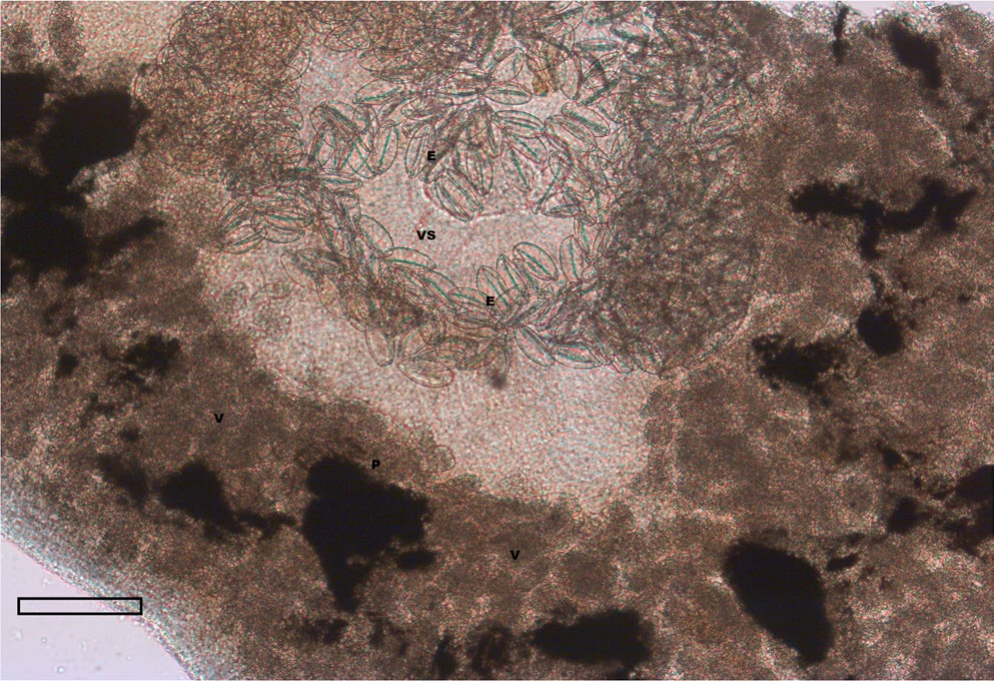
Numerous eggs in uterus above the ventral sucker of *C. oblonga*. E: Egg, VS: Ventral sucker. Scale bar: 200 μm

**Fig. 5:**
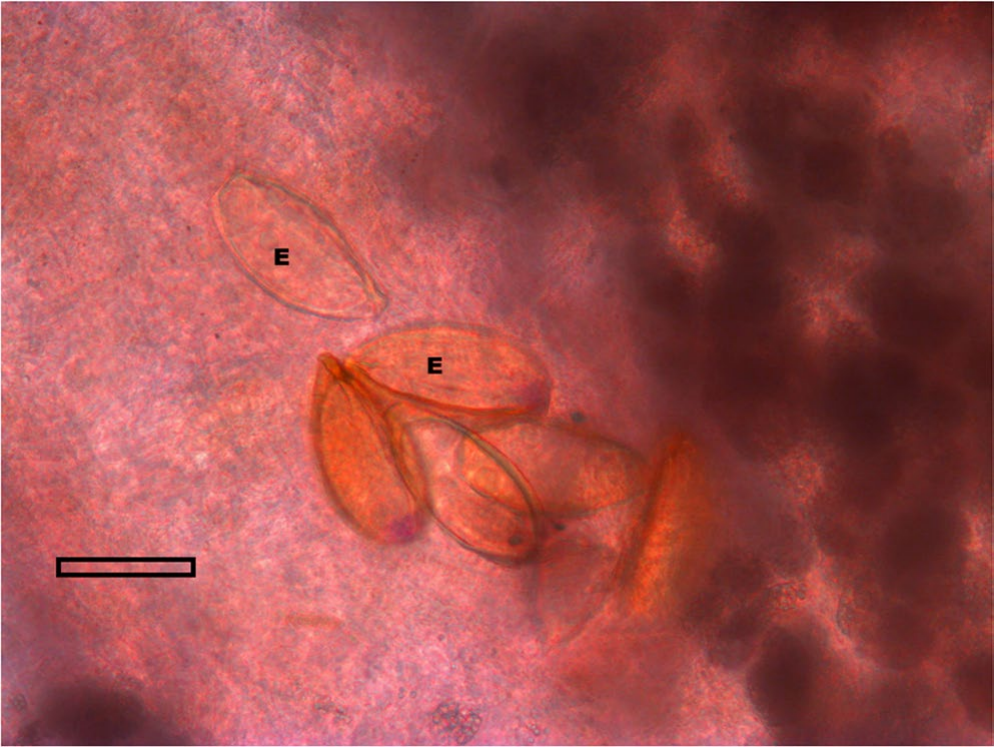
Fraction of eggs isolated along the pharynx of *C. oblonga.* E: Egg. Scale bar: 60 μm

**Fig. 6:**
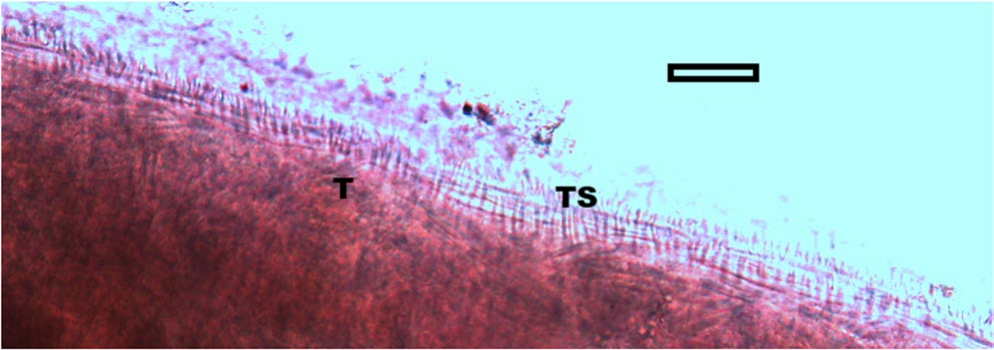
Dense tegumental spine covering of the *C. oblonga* anterior part. TS: Tegumental spines. T: Tegument. Scale bar: 30 μm

The morphological analysis (Figs. 1, 2, 3, 4 and 5) and morphometric characteristics of the Danish isolate (Table 1) complied in most cases well with descriptions provided by Cobbold (1858). He described trematodes (probably at different developmental stages and non-compressed) with body length 3.175 to 6.35 mm and width 1.7 mm, whereas our full grown and flattened specimens measured from 6.9 to 7.9 mm (total length) and width from 1.9 to 2.1 mm. Apart from this discrepancy oral (Fig. 2) and ventral suckers were conspicuous and the reproductive pore located in front of the ventral sucker, exactly as in the original description. The tegument was found equipped with a dense layer of minute spines (Fig. 6) as reported by Cobbold (1858). However, the density of spines was in the present isolates higher than illustrated by Cobbold (1858). Vitellaria were in our specimens, as in the original type species, largely developed. In the stained specimens the vitellaria were opaque and covered the intestine. The intestinal caeca described an irregular zigzag form. The accompanying drawing by Cobbold (1858) showed less developed vitellaria (probably a young specimen) but a conspicuous zigzag shaped intestine and an elongated pharynx. This characteristic form of the intestine was in full agreement with our non-stained Danish isolate, in which vitellaria had been cleared (in glycerine and glycerine-gelatine) and made partly transparent, whereby the intestinal caeca with contents became highly visible (Figs. 2 and 3). Eggs were not considered in the original description, but we found numerous eggs (length 75–90 μm, width 45–50 μm) in the uterus (Fig. 4) and a few trapped along the pharynx in several specimens (Fig. 5). The egg-filled uterus was located at the ventral sucker position (Figs. 1 and 2). Later studies on the species recovered from harbour porpoise in the Western part of the Atlantic Ocean were presented by Price (1931) and Adams et al. (1998). Other datasets on the species are available from Pacific isolates. A few are *C. oblonga* isolated from harbour porpoise but others are from other hosts such as *Phocaenoides dalli* True, 1885. Those isolates were described by Yamaguti (1951). Corresponding *C. oblonga* parasites were recovered from the narrow-rigded finless porpoise *Neophocaena asiaeorientalis* Pilleri and Gihr, 1972 and described by Kuramochi et al. (2000) and Kim et al. (2025). When comparing *C. oblonga* data from *P. phocoena* (present study and West Atlantic isolates) with Pacific isolate data from *N. asiaeorientalis* (Table 1) differences are suggested. Especially total length, ventral sucker length and width and pharynx length tend to be larger in Pacific isolates. Thus, body length in the Pacific isolates is up to 10.5 mm and body width is up to 3.12 mm compared to our Atlantic porpoise parasites showing body length up to 7.9 mm and width up to 3.0 mm. Corresponding differences were noted for pharynx and ventral sucker dimensions (Table 1). However, due to the low number of specimens examined, individual variation of parasites and possible effects of slide preparation (compression artefacts), possible differences between Atlantic and Pacific isolates should be further investigated and valid statistical tests performed.

**Table 1:** Comparative listing of *C. oblonga* morphometric details (mean and range). Atlantic isolates from harbour porpoise *P. phocoena* compared to Pacific isolates from *N. asiaeorientalis*. *: Data from Kim et al. (2025) as original reference was un-accessible. Rows in bold indicate possible differences. #: Only range available

Reference	Present study	Price (1931) #	Adams et al. (1998)	Kim et al. (2025)	Kuramochi et al. (2000) * #
Geographic origin	North Sea coast of Denmark	West Atlantic North America	West Atlantic, Washington, North America	Korean Southern Sea	Japanese seas
Host species	*Phocoena phocoena*	*Phocoena phocoena*	*Phocoena phocoena*	*Neophocoena asiaeorientalis sunameri*	*Neophocoena asiaeorientalis sunameri*
Number examined	4	4	10	15	20
Body length mm	**7.35 (6.90–7.90)**	**4.00–7.00**	**7.29 (6.61–7.78)**	**7.59 (7.03–8.66)**	**8.23–10.5**
Body width mm	2.03 (1.90–2.10)	1.00–3.00	2.1 (1.80–2.28)	2.38 (2.05–2.66)	2.29–3.12
Oral sucker length µm	315 (290–350)	310–340	307 (286–330)	283 (227–321)	270–390
Oral sucker width µm	340 (300–380)	310–340	307 (297–319)	309 (280–334)	300–320
Ventral sucker length µm	**450.3 (401–500)**	**430–465**	**429 (385–473)**	**556 (496–640)**	**500–640**
Ventral sucker width µm	**447.5 (400–500)**	**430–465**	**424 (396–462)**	**604 (527–656)**	**600–680**
Pharynx length µm	**267.5 (300–430)**	**310–360**	**359 (319–396)**	**417 (356–477)**	**440–500**
Pharynx width µm	170 (150–190)	170–220	191 (165–209)	157 (122–199)	210–250
Ovary length µm	207.5 (180–240)	186–372	372 (286–429)	320 (256–357)	360–420
Ovary width µm	277.5 (200–350)	310–527	410 (308–506)	435 (299–516)	420–650
Anterior testes length µm	925 (790–1210)	620–770	894 (737–1000)	786 (608–934)	840–1220
Anterior testes width µm	1250 (900–1400)	770–990	1,122 (968-1,320)	1092 (879–1292)	1040–1500
Posterior testes length µm	1210 (840–1500)	620–1000	1,142 (946-1,298)	1050 (830–2141)	946–1298
Posterior testes width µm	1125 (1000–1300)	770–1200	1,082 (891-1,221)	1081 (878–1279)	830–1430
Eggs length µm	85 (75–90)	90–97	86 (79–95)	69 (51–80)	72–84
Eggs width µm	48.8 (45–50)	44–54	45 (41–49)	37 (28–45)	45–50

### Molecular identification

**Fig. 7:**
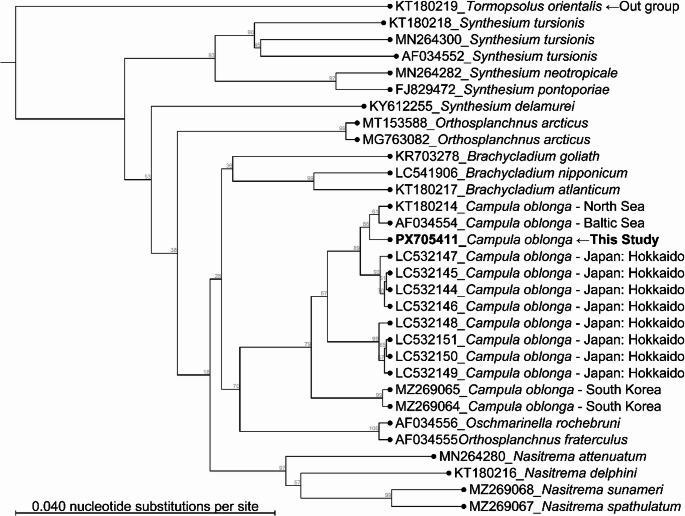
Phylogenetic analysis of the ND3 sequence obtained from *C. oblonga* isolated from harbour porpoise in Denmark

The mitochondrial gene encoding NADH dehydrogenase subunit 3 (ND3) was amplified by PCR, the 473 bp long product purified and sequenced. The sequence was submitted to GenBank and obtained accession no. PX705411. The phylogenetic analysis (Fig. 7) clearly indicated a closer relationship between our North Sea isolate and previous findings in the North Sea and the Baltic Sea. It consisted of the partial tRNA-Leu (leucine) (bp – to 47), the tRNA-Lys (lysine) (bp 58 to 123), and partial NADH dehydrogenase subunit 3 (ND3) (bp 127 to 473). The ND3 part was subjected to BLAST search at GenBank (January 6, 2026) and revealed highest identity toward isolates of *Campula oblonga* sequences, e.g. GenBank accession no. KT180214 isolated from the North Sea in the host *Phocoena phocoena* (identity 99.11%) and *Campula oblonga* sequences GenBank accession no. AF034554 isolated from the host *Phocoena phocoena* from the Baltic Sea in (identity 98.27%). The similarity between our Atlantic ND3 sequences and the Pacific *C. oblonga* sequences was much lower. Thus, the Korean sequences MZ26904 and MZ26905 showed identities of 93.35% and 93.66%, respectively. The similarity to other species within the same family was low: *Oschmarinella macrorchis* (LC326064) with the identity 91.64% and *Orthosplanchnus fraterculus* (AF034555) with the identity 90.78%. The phylogenetic analysis including available *C. oblonga sequences* of the ND3 sequence (Fig. 7) supported the BLAST search result and further indicated that the Pacific isolates phylogenetically were less related to the Atlantic forms.

## Discussion

The digenean *C. oblonga* in the family Brachycladiidae (subfamily Brachycladiinae) Odhner, 1905 was originally isolated from a harbour porpoise *P. phocoena*, which was shot in the Firth of Forth (East coast of Scotland connected to the North Sea) in April 1855. It was described by Cobbold (1858), reporting that the location of the adult trematodes in the peripheral bile-ducts was associated with enlargement and thickening of the duct walls. Corresponding reactions were found as periductular fibrosis in infected hosts from the Baltic Sea (Dzido et al. 2021) and in the coastal areas of Korea (Kim et al. 2025). The present study confirmed that *C. oblonga* parasites were associated with chronic hepatitis and cholangitis. Thus, the pathologically affected parts of the liver were fibrotic with abscesses, characterized by ectasia, enlarged and cavernous bile ducts. It may be hypothesized that the numerous tegumental spines exert a mechanical disruption of the epithelia covering the bile duct wall, which subsequently would elicit an inflammatory reaction. The parasite may benefit from the spines as they allow the parasite to remain attached at a certain site in the bile duct despite a steady flow of bile. The taxonomy of the species has been analyzed by Skrjabin (1948) and Gibson (2005). It has been redescribed by various authors including Price (1931), Yamaguti (1951) and reported from various locations such as coastal Korea (Kim et al. 2025). A number of investigations have been performed on the parasites recovered from harbour porpoise specimens found dead along the North Sea coasts and it is well documented that a wide range of helminths parasitizes these cetaceans and among these *C. oblonga* have been reported from the UK (Gibson et al. 1998), Germany (Siebert et al. 2001), Poland (Rokicki et al. 1997; Dzido et al. 2021) and Norway (Lehnert et al. 2005). However, species identification based on molecular techniques were applied merely in few studies. Especially the gene encoding the isoform ND3 of the mtDNA has been targeted in these studies (Fernández et al. 1998; Fraija-Fernández et al. 2016; Kim et al. 2025). The infected porpoise, which was the basis of the present investigation, corresponded to the type host from the Firth of Forth. It was found dead on the North Sea coast of Denmark and thereby geographically closely connected to the type locality, which makes it likely that we here have specimens closely related to the original isolates. The morphometric and morphological characteristics corresponded very well with the original description and especially the zigzag shaped intestinal caeca, specifically addressed and drawn by Cobbold (1858), were prominent in the cleared but non-stained isolate. The vitellaria in this trematode species are very well developed and in the stained specimens covered the intestine and other organs, whereby the special morphology of the intestine may not be readily seen in some whole mount slides, as noted by Gibson (2005). Thus, the line drawings presented by Price (1931), Yamaguti (1951), Gibson (2005) and Kim et al. (2025) do not clearly show the characteristic intestinal morphology because the vitellaria in those specimens are well developed and covering the characteristic intestinal shape. By specifically clearing our specimen we were able to illustrate the intestinal morphology. The trematode *C. oblonga* has been reported from cetaceans by a number of other authors (Jaber et al. 2004; Rokicki et al. 1997; Dzido et al. 2021), but the species diagnosis was in those cases not associated with line drawing, photos or molecular sequence information. In this work we present both morphometric and molecular details and compare them with previously reported data. We showed that our isolate complied with existing data of *C. oblonga* isolated in the Atlantic region. There has been some confusion about testes morphology (Gibson 2005). The very dominating and branching vitellaria may in whole mounts make the testes look lobed, but in the Danish isolate the testes outlines were smooth and unlobed. This is a differential characteristic compared to the Pacific isolate presented as line drawing by Kim et al. (2025). The body dimensions were within the range seen with other specimens of *C. oblonga* from *P. phocoena* in the Atlantic (Price 1931; Adams et al. 1998), but it was noted that *C. oblonga* specimens obtained from other host species in the Far East differed slightly. Thus, the total length, ventral sucker and pharynx length tended to be larger in the liver trematodes recovered from *N. asiaeorientalis sunameri* (Kim et al. 2025; Kuramochi et al. 2000). However, due to a limited number of measurements, individual size variation of parasites, host effects, and slide preparation technique (compression effect) size differences should be treated with caution when differentiating species. Nonetheless, we supplemented the morphometric and morphological characterization with a molecular analysis focusing on the mtDNA gene encoding NADH (isoform ND3). It proved to be well suited for species differentiation. This region showed highest similarity with the ND3 sequences obtained from *C. oblonga* recovered from the North Sea (Fraija-Fernández et al. 2016). Corresponding and closely related sequences of *C. oblonga* isolated from harbour porpoise from the Baltic Sea were presented by Fernández et al. (1998), but these authors did not report the precise geographic location of the host and morphological details were not presented. The ND3 sequences presented for *C. oblonga* isolated from *P. dalli* in Japan (Nakagun and Kobayashi 2020) and *N. asiaeorientalis* in Korean waters (Kim et al. 2025) proved to be phylogenetically far more distant. Further extended investigations (both morphometric and molecular) should therefore elucidate if the recorded morphometric, morphological and molecular dissimilarities demonstrated in the present study will allow subdivision of *C. oblonga* into Pacific and Atlantic subspecies. The life cycle has not been fully elucidated. We hypothesize that a marine mollusc species is the first intermediate host releasing cercariae. Stomach analyses of whales suggest that both fish and cephalopods may serve as second intermediate hosts and carry the metacercarial stage of brachycladiids (Nakagun et al. 2018). We acknowledge that field sampling and investigation of life cycles are extremely laborious. However, in order to achieve clarification of this ecological central question we recommend implementation of systematic investigations of parasitofaunas in potential intermediate host groups.

## Data Availability

All data are available in the submitted manuscript file.
